# Modulation of COVID-19 incidence by environmental stressors is variant between pre-Omicron and Omicron periods

**DOI:** 10.1038/s41598-025-13521-2

**Published:** 2025-07-29

**Authors:** Leona Hoffmann, Lorenza Gilardi, Tobias Antoni, Maxana Baltruweit, Michael Bittner, Susanne Breitner, Simon Dally, Thilo Erbertseder, Sabine Hawighorst-Knapstein, Marie-Therese Schmitz, Rochelle Schneider, Sabine Wüst, Jörn Rittweger

**Affiliations:** 1 Institute of Aerospace Medicine, German Aerospace Center (DLR), Cologne, Germany; 2 German Remote Sensing Data Center, German Aerospace Center (DLR), Weßling, Germany; 3 Allgemeine Ortskrankenkasse Baden-Württemberg (AOK-BW), Stuttgart, Germany; 4 Institute of Epidemiology, Helmholtz Zentrum München - German Research Centre for Environmental Health, Neuherberg, Germany; 5 IBE-Chair of Epidemiology, LMU Munich, Munich, Germany; 6 Institute of Medical Biometry, Informatics and Epidemiology (IMBIE), University Hospital Bonn, Bonn, Germany; 7 Φ-Lab, European Space Agency (ESA), Frascati, Italy; 8 Department of Pediatrics and Adolescent Medicine, University Hospital Cologne, Cologne, Germany

**Keywords:** Environmental sciences, Respiratory tract diseases, Epidemiology

## Abstract

COVID-19 had a devastating impact on humanity. We investigated how residential air pollution (ozone (O_3_), nitrogen dioxide (NO_2_), fine particulate matter (PM_2.5_)) and meteorological factors (temperature (Temp), precipitation (Prec)) are associated with COVID-19 incidence in Baden-Württemberg (BW), Germany. We utilized data from the Copernicus Atmosphere Monitoring Service and the Copernicus Climate Change Service to model environmental exposure from 2020 to 2022 in postal code areas in BW. Health insurance data on SARS-CoV-2 infections were provided from the health insurance AOK BW on a quarterly level covering approximately 12 million person-years. We examined the spatiotemporal variability with a generalized additive model including various stressors, demographic factors, and area-wide data, offering a comprehensive analysis of the environmental stressor- COVI-10 incidence associations. In 2022, during the prevalence of the Omicron variant, the number of COVID-19 cases tripled compared to 2020. During the pre-Omicron period, COVID-19 incidence showed a positive association with PM_2.5_ (relative risk [RR] 2.41; 95% confidence interval [CI] (2.31, 2.52)), a negative association with Temp (RR 0.39 (0.32, 0.48)), and no clear or slight associations with O_3_, Prec, and NO_2_. During the Omicron period, there were either no clear or slight negative associations with Temp (RR 0.92 (0.74, 1.30)), PM_2·5_ (RR 0.70 (0.64, 0.79)), NO_2_, and Prec and a negative association with O_3_ (RR 0.46 (0.40, 0.53)). The analysis found clear links between environmental stressors and COVID-19 incidence, which strongly differed between pre-Omicron and Omicron periods. Consideration of environmental stressor concentration could be relevant in the management of the pandemic.

## Introduction

The COVID-19 pandemic has been a fatal disruption at the start of the twenty-first century, with long-reaching consequences. From 2020 to 2022 there were 732 million COVID-19 cases globally, 269 million in Europe, and 37 million in Germany (status: January 1, 2023)^1^. Ambient air pollution is a major risk to human health, with fine particulate matter of diameter ≤ 2.5 µm (PM_2.5_) and nitrogen dioxide (NO_2_) being prominent contributors. Several previous studies have reported associations between environmental stressors and COVID-19 infections^2,3^. However, most scientific papers on this topic were published in the early days of the pandemic, thus with relatively short observation interval, and with a focus on densely populated areas^4–6^. Review articles have summarized the environmental impact on COVID-19 peaks in 2020 and 2021 for various regions worldwide, including Asia, Europe, America, and the Middle East^7^, concluding that exposure to air pollution can facilitate COVID-19 transmission^2,8–12^. More specifically, positive associations with COVID-19 incidence were repeatedly reported for PM_2.5_ and NO_2_^2,3,7–9^, and a negative association with temperature^13–15^ . For a comprehensive assessment of environmental effect modulations, it is therefore necessary to analyze a more extended time span, covering the entire 2020–2022 period.

Moreover, the emergence of COVID-19 variants has introduced major antigenic changes to the SARS-CoV-2 virus, with implications for transmissibility, and infection dynamics^16^. These changes have likely influenced not only viral transmissibility and clinical severity, but also the interaction between the virus and external factors^17^, including environmental exposures. The Omicron variant, in particular, spread rapidly despite high levels of population immunity^18^, suggesting altered infection dynamics and transmission patterns. We therefore hypothesized that the associations between environmental exposures, such as PM₂.₅, NO₂, and temperature, and COVID-19 incidence may differ between the pre-Omicron and Omicron periods and conducted a stratified analysis accordingly.

Previous scientific studies have often concentrated on particular urban areas, such as Milan (Italy)^5^, Wuhan (China)^19^ or Vienna (Austria)^4^, or on particular environmental stressors, like NO_2_^20,21^, and particulate matter^22,23^. Urban and rural areas differ in pollution levels, and also in the spread and course of diseases. Our study covered the entire federal state of Baden-Württemberg (Germany), thus including both urban and rural regions and thereby offering a better basis for generalizable findings than spatially selective studies. Moreover, air pollutions have been shown to interact with meteorological factors. For example, ambient temperature and PM_2.5_ can jointly explain seasonal modulation of influenza incidence^24^. The relationships between air pollution parameters and meteorological conditions are complex and not always obvious^25^, offering potential for misinterpretation when environmental stressors such as PM_2.5_ are studied stand-alone. A comprehensive understanding also requires a comprehensive set of observations^11^. There is a need to include confounding factors such as age, sex assigned at birth, and population density in the analysis^8,9,26^.

Therefore, the present paper presents a comprehensive analysis of the association between COVID-19 incidence and environmental stressors in Baden-Württemberg, Germany, between 2020 and 2022, stratified by the pre-Omicron and Omicron periods.

## The pandemic in Baden-Württemberg

In March 2020, the World Health Organization declared Europe an epicenter of the pandemic, leading to border closures and strict restrictions on public life. Member states implemented contact and exit restrictions to control the spread of the virus. Baden-Württemberg is a federal state in southwestern Germany with a population of 11.28 million, making it more populous than some European countries like Austria or Finland^27^. It is a diverse state with urban, rural, and mountainous regions. The COVID-19 pandemic significantly impacted Baden-Württemberg due to its dynamic pandemic activity. The government implemented several restrictive measures to control the pandemic’s spread, including mandatory facial masks, social distancing, school closures, travel and restaurant restrictions, and public and private gatherings limits.

**Fig. 1:**
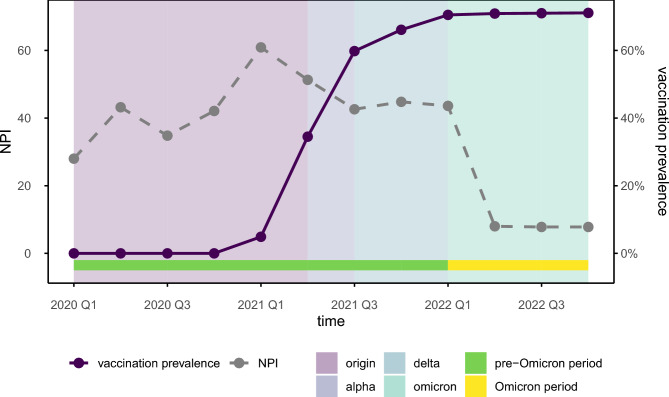
Illustration of severe acute respiratory syndrome coronavirus 2 (SARS-CoV-2) variants, non-pharmaceutical intervention (NPI) and vaccination prevalence in Baden-Württemberg. Vaccination data are aggregated as quarterly means. Reference population for BW as of Dec 31, 2022, is 11.28 M people^34^. SARS-CoV-2 variants are classified quarterly based on the prevailing virus strain. As no specific SARS-CoV-2 variants data are available for BW, it is assumed that the variants behaved similarly as in the rest of Germany. Using the NPI, we could track and observe the government’s responses to COVID-19 over time in Baden-Württemberg.

The German Federal Ministry for Economic Affairs and Climate Action has commissioned the creation of a non-pharmaceutical intervention (NPI) to evaluate COVID-19 measures implemented continuously since March 1, 2020^28^. This index, which is provided by infas 360^29^, is based on the Oxford COVID-19 Government Response Tracker^30^, and amalgamates the various restriction types at any given time into a single score value. The NPI is based on 21 key areas of public life, including gatherings, schools, childcare, events, cultural institutions, retail, nightlife, accommodations, sports, travel restrictions, mask mandates, workplace regulations, curfews, public transport capacity, distancing rules, and testing policies. Each area was coded with specific measures ranked on a scale from 0 (“no restriction”) to higher values for more stringent interventions. To make it compatible with our analysis, we aggregated the monthly information as quarterly mean values. The NPI index peaks in Q1 of 2021 (Fig. 1).

The COVID-19 vaccination campaign in Germany began on December 27, 2020. Initially, high-risk individuals and those with occupational exposure were prioritized, until the vaccine eventually became available to everybody in June 2022^31^. The basic immunization rate reached 71% of the BW population by the end of 2022, with the largest increase observed in Q2 and Q3 of 2021 (Fig. 1). Although it was initially assumed that two vaccinations suffice for basic immunization, infections occurred frequently despite vaccination^18^. Information on German COVID-19 vaccination is available from Zenodo^32^, while information on SARS-CoV-2 variants can be retrieved from the weekly dashboard of the Robert Koch Institute (RKI)^33^. The original SARS-CoV-2 variant dominated up to and including Q1/2021, to be replaced by the Alpha variant in Q2/2021, the Delta variant in Q3/2021, and the Omicron variant in Q1/2022 (Fig. 1).

## Materials and methods

### Health data

The Allgemeine Ortskrankenkasse Baden-Württemberg (AOK BW) is one of Germany’s largest health insurance companies, operating since more than 130 years, and covering over 4.6 million people. Our data include the number of COVID-19 infections and the total number of insured persons from 2020 to 2022, aggregated by quarter, postal code area, sex assigned at birth, and the age group in 10-year increments where the numerical value corresponds to the age of the oldest person in the group. As in our previous study^24^, the data sources within the AOK BW electronic system were outpatient and inpatient hospital data, sick-leave notes and outpatient diagnoses within the framework of home and specialist centered care and standard care. COVID-19 cases were identified on a quarterly basis via the International Classification of Diseases 10^th^ Revision (ICD-10) codes U07.1 (COVID-19, virus detected) and U07.2 (COVID-19, virus not detected). Using these data sources ensures maximum sensitivity when identifying infected person groups. All methods were conducted by relevant guidelines and regulations. A positive ethics vote and a waiver of informed consent was obtained from the North Rhine Medical Association (number: 2020092), and data processing was approved by AOK BW and DLR. The incidence estimates are based on data from AOK Baden-Württemberg, which covers a large and demographically representative segment of the regional population. Internal analyses by AOK indicate similarity in key factors such as age, sex, and morbidity between the insured and the general population in BW. Nonetheless, incidence estimates apply specifically to the insured group, and caution is advised when generalizing to the entire population.

### Environmental data

Surface-level data on outdoor air pollution, including NO_2_ (in µg/m^3^), ozone (O_3_, in µg/m^3^), and PM_2.5_ (in µg/m^3^)^35^, based on the Copernicus Atmosphere Monitoring Service (CAMS) Air Quality Reanalysis dataset^36^. The meteorological data precipitation (Prec, in mm/day) and temperature (Temp, in °C) is sourced from the ERA5-Land, a reanalysis dataset provided by the Copernicus Climate Change Service (C3S) of the European Centre of Medium-Range Weather Forecasts (ECMWF)^37^. Both initial datasets have a 0.1° × 0.1° spatial and hourly temporal resolutions. To address the spatial mismatch between the coarser environmental datasets and the finer scale of postal code areas, we applied bilinear interpolation to oversample the spatial resolution of all variables. Based on the interpolated data, daily mean values were computed for each grid cell, and spatial aggregation was then performed by calculating the mean across all grid cells intersecting each postal code area defined by a polygon, following established recommendations^38,39^. For ozone (O₃), the daily maximum of the 8-h rolling mean was calculated in line with WHO air quality guidelines^40^. Finally, all daily postal code-level estimates were averaged to the quarterly level for the years 2020 to 2022 to align with the temporal resolution of the health data.

### Data processing

The health and environmental data sets were processed and analyzed using R version 4.3.0^41^ and combined using the `merge()` function from R’s base package. This merging process was based on three key variables: PLACE, which serves as the geographic identifier for postal code areas; YEAR, representing the calendar year; and QUARTER, indicating the quarter of the year. This approach ensured accurate temporal and geographic alignment of the two data sets. We utilized a shapefile containing five-digit postal codes sourced from the Esri Germany database to manage postal code areas effectively^42^.

### Statistical methods

**Fig. 2:**
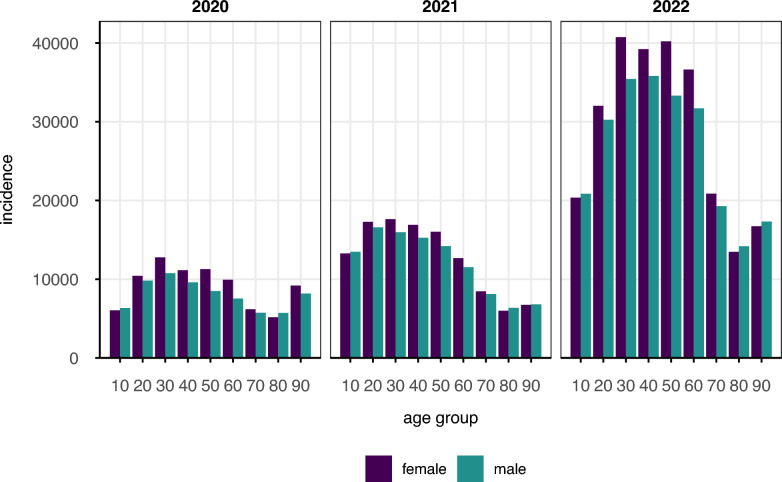
COVID-19 incidence categorized by year, age, and sex assigned at birth. The incidence was defined as the number of COVID-19 cases per 100 thousand AOK-insured individuals per year.

**Fig. 3:**
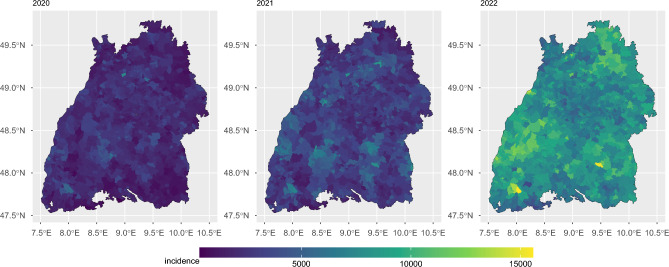
Spatial distribution of COVID-19 incidence by postal code area faceted by year. The incidence was defined as the number of COVID-19 cases per 100 thousand AOK-insured individuals per year.

We generated descriptive statistics, categorizing COVID-19 incidence by year, sex assigned at birth, and age (Fig. 2), and with incidence maps for each year (Fig. 3). The overall infection rates by sex were calculated, along with 95% confidence intervals, using R’s binom.test() function. In addition, we utilized a generalized additive model (GAM)^43^, as previously^24^, to investigate the associations between environmental stressors and COVID-19 incidence. The model was implemented using the bam() function from the mgcv package in R^44^, employing penalized maximum likelihood estimation (REML) for smoothing parameter selection. The dependent variable was the number of new COVID-19 cases, modeled with a negative binomial distribution. The explanatory variables included both linear and nonlinear terms. We fitted smooth functions with penalized splines for the environmental stressors Temp, PM_2.5_, NO_2_, O_3_, and Prec^45^. The categorical variables age group, gender, quarter and year were included as fixed effects to address temporal effects and demographic information. To account for spatial variation, we incorporated a Markov random field (MRF) smooth term for postal code areas (bs = “mrf” in bam()), where the neighborhood structure was automatically constructed from shared polygon boundaries provided via the polygon list^43,44^. The smoothing basis for this term was restricted to 20 dimensions. We included an offset for the number of AOK-insured persons per five-digit postal code to adjust for population density and other features linked to post code (e.g. socio-economic status). A stratified subgroup analysis was performed for the pre-Omicron and Omicron periods by run separate GAMs for each period. We defined the incidence as the number of COVID-19 cases per 100 thousand AOK-insured individuals per year. per year. Based on the GAMs we estimated the incidence depending on the individual environmental stressors and used R’s predict() function. The datasets were split into a training set (70%) and a test set (30%) to evaluate model performance. The split was balanced across postal code area and year to preserve the temporal and spatial structure of the data. Model performance was assessed by comparing observed and predicted values^46^. To quantify the impact of the environmental stressors, the relative risk (RR) was calculated as the 95%-to-5% ratio of the environmental stressor predictions, following our previous approach^24^. The 95% confidence intervals for the RR values were calculated using the percentile bootstrap method with 1000 repetitions for each model^46^. To test the robustness of results, we conducted sensitivity analyses excluding the top 1% of extreme values for each air pollutant.

## Results

### Descriptive results

Between 2020 and 2022, the AOK BW reported more than 2 million COVID-19 infections. The incidence rose over the years, with about 10,000 new infections per age group per 100,000 persons reported in 2020, about 15,000 in 2021, and over 30,000 in 2022, indicating that COVID-19 incidence has tripled in 2022 compared to 2020 (Fig. 2). The overall infection rate for females was 53.24% [95% confidence interval (CI): 53.17%, 53.30%] and for males 49.76% [95% CI: 49.69%, 49.83%].

As illustrated in Fig. 3, the geographical distribution of the year-wise increase in incidence showed no clear distinction between urban and rural areas. In 2022, two outstandingly high incidences (yellow spots) were reported in Bernau im Schwarzwald (postal code 79872) and Ertingen (postal code 88521).

The inter-relationships among the various environmental stressors in Baden-Württemberg were discussed previously^25^. The Supplementary Material provides a detailed table of the concentration levels of the environmental stressors (Table S1) and correlation matrices (Figure S1) specifically for the years 2020 to 2022.

### GAM results

**Fig. 4:**
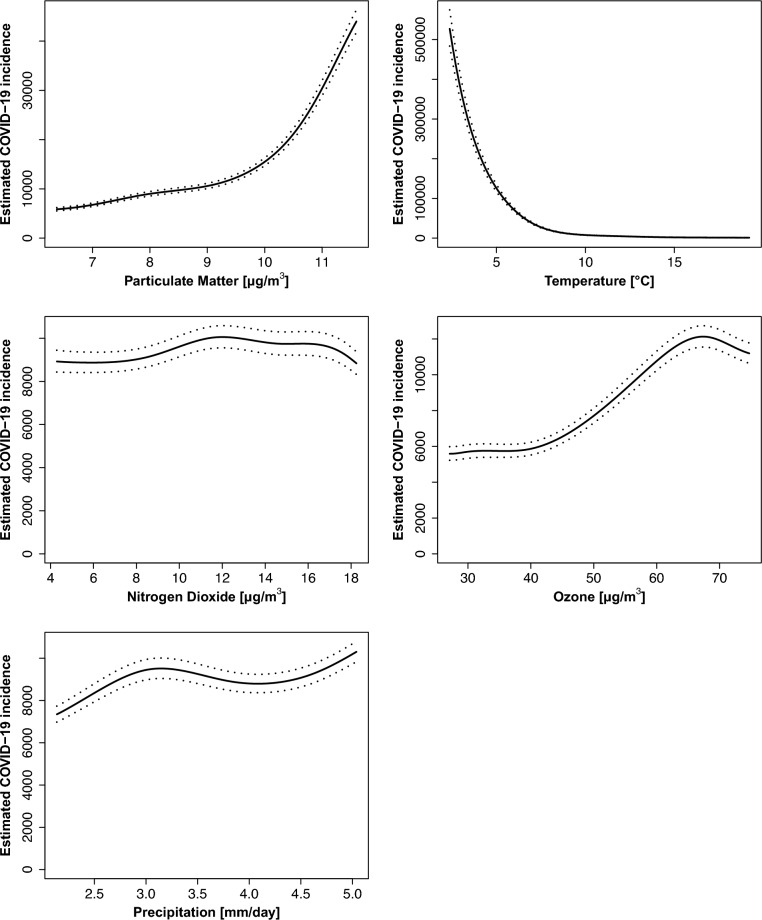
Estimated COVID-19 incidence per 100,000 persons per year in response to each environmental stressor. All other parameters were kept constant, and were set to the following values: Age group: 50–60 years, sex assigned at birth: female, postal code: 70376, quarter: Q2, year: 2021 and environmental stressors: median. Estimates were obtained using the R function predict for Temp, Prec, NO_2_, O_3_, and PM_2.5_, including their 5th and 95th percentiles.

Whilst PM_2.5_ (RR 6.27, 95% confidence interval CI (6.05, 6.50), Table 1) and O_3_ (RR 2.04 (1.94, 2.16), Table 1) were positively associated with COVID-19 incidence, ambient Temp (RR 0.003 (0.003, 0.004), Table 1) was negatively associated (Fig. 4). No clear-cut or weak associations were found for NO_2_ (RR 1.05 (1.01, 1.10), Table 1) and Prec (RR 1.29 (1.26, 1.33), Table 1).

**Table 1:** Estimated COVID-19 incidence and associations to environmental stressors during different periods of the pandemic.

	Output	COVID-19 Overall	pre-Omicron period	Omicron period
PM_2·5_	Prediction 5-Percentile Prediction 95-Percentile **Relative Risk**	6072.98 (5745.25, 6419.41) 38,085.95 (36,176.79, 40,095.87) **6.27 (6.05, 6.50)**	2548.32 (2322.88, 2795.63) 6136.83 (5550.48, 6785.12) **2.41 (2.31, 2.52)**	17,571.66 (12,571.68, 19,723.30) 12,304.79 (10,147.77, 14,920.32) **0.70 (0.64, 0.79)**
NO_2_	Prediction 5-Percentile Prediction 95-Percentile **Relative Risk**	8892.68 (8417.80, 9394.36) 9351.00 (8816.68, 9917.69) **1.05 (1.01, 1.10)**	3121.89 (2835.20, 3437.58) 2188.84 (1982.89, 2416.17) **0.70 (0.66, 0.74)**	16,372.97 (13,591.75, 19,723.30) 12,701.18 (10,524.07, 15,328.68) **0.78 (0.70, 0.83)**
O_3_	Prediction 5-Percentile Prediction 95-Percentile **Relative Risk**	5653.11 (5287.54, 6043.96) 11,538.9 (10,982.98, 12,122.97) **2.04 (1.94, 2.16)**	4255.69 (3840.01, 4716.37) 4263.45 (3873.63, 4692.50) **1.00 (0.93, 1.08)**	27,664.95 (22,033.03, 34,736.47) 12,696.59 (10,628.35, 15,167.29) **0.46 (0.40, 0.53)**
Temp	Prediction 5-Percentile Prediction 95-Percentile **Relative Risk**	361,166.50 (332,729.00, 392,034.40) 1232.60 (1154.46, 1316.03) **0.003 (0.003, 0.004)**	12,184.78 (10,552.70, 14,069.29) 4785.45 (4326.25, 5293.39) **0.39 (0.32, 0.48)**	15,975.74 (12,630.65, 20,206.74) 14,769.59 (13,639.19, 15,993.68) **0.92 (0.74, 1.30)**
Prec	Prediction 5-Percentile Prediction 95-Percentile **Relative Risk**	7653.14 (7270.31, 8056.12) 9896.35 (9441.55, 10,373.05) **1.29 (1.26, 1.33)**	2925.48 (2658.21, 3219.62) 3927.30 (3601.46, 4282.63) **1.34 (1.29, 1.39)**	16,543.51 (13,761.47, 19,887.97) 14,109.23 (10,628.35, 16,905.05) **0.85 (0.83, 0.88)**

**Fig. 5:**
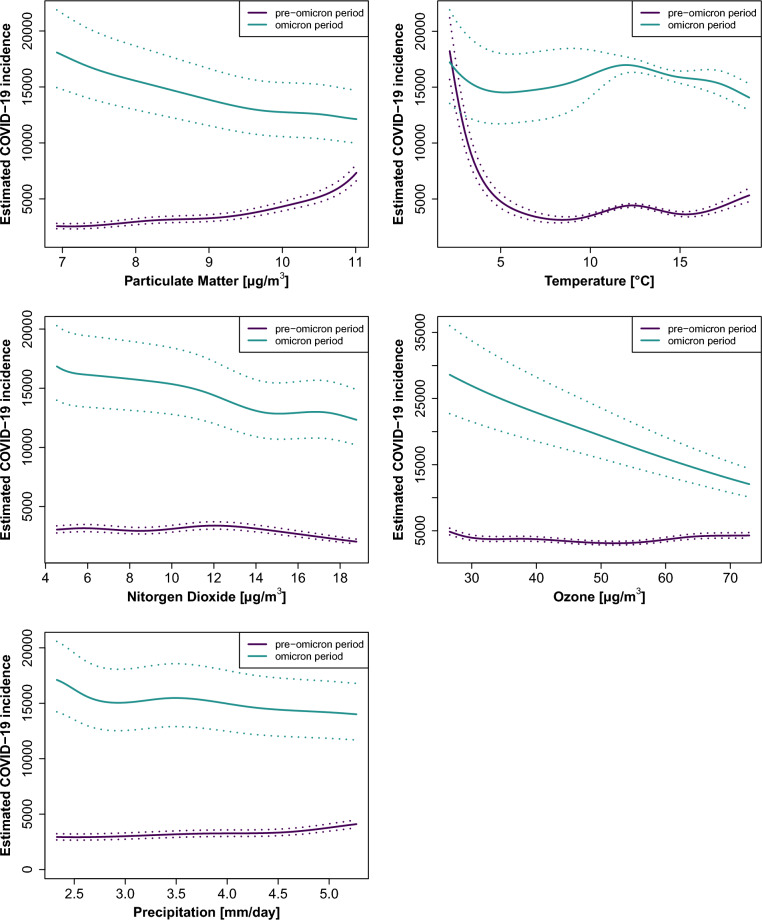
Estimated COVID-19 incidence per 100,000 persons per year in response to each environmental stressor based on pre-Omicron (2020, 2021, purple) and Omicron (2022, teal) model. All other parameters were kept constant and were set to the following values: quarter: Q2, year (optional): 2021, postal code: 70376, sex assigned at birth: female, age group: 50 to 60 years, and environmental stressor: median. Estimates were obtained using the R function predict for Temp, Prec, NO_2_, O_3_, and PM_2.5_, including their 5th and 95th percentiles.

The table enlisted the estimated number of cases per 100,000 persons per year for 5% and 95% percentiles of the environmental stressors split by the overall model (Fig. 4) and the pre-Omicron and Omicron period (Fig. 5). The values of the confidence interval are given in brackets. Green cells indicated RR > 1.3 (strong positive association), yellow cells RR < 0.7 (strong negative association), and uncolored cells 0.7 ≤ RR ≤ 1.3 (weak or no association). The incidence was defined as the number of COVID-19 cases per 100 thousand AOK-insured individuals per year.

When comparing pre-Omicron and Omicron periods, the relationships revealed a different picture (Fig. 5). Whilst associations for the pre-Omicron period were very similar to the overall model (Fig. 4), the Omicron period showed a negative association of O_3_ (RR 0.46 (0.40, 0.53), Table 1) and a slight negative association of PM_2.5_ (RR 0.7 (0.64, 0.79), Table 1) and NO_2_ (RR 0.78 (0.70, 0.83), Table 1) with COVID-19 incidence and no clear-cut associations for Temp (RR 0.92 (0.74, 1.30), Table 1) and Prec (RR 0.85 (0.83, 0.88), Table 1) concentrations.

According to our GAM models, the expected number of COVID-19 cases was 8–9% lower for males than for females in all three COVID-19 models (RR Overall: 0.92 (0.91, 0.92); RR Omicron: 0.91 (0.91,0.92); RR pre-Omicron: 0.92 (0.91, 0.92)). Comparing 2022 to 2020, the expected number of cases increased by a factor of 3.97 for the COVID-19 overall model. Compared to the 50–60 age group, the expected number of new COVID-19 cases was lower for the 70–80 age group (RR Overall: 0.49 (0.48, 0.50); RR Omicron: 0.40 (0.39, 0.41); RR pre-Omicron: 0.57 (0.65, 0.58)) and higher for the 20–30 age group (RR Overall: 1.23 (1.21,1.24); RR Omicron: 1.04 (1.03, 1.06); RR pre-Omicron: 1.38 (1.36, 1.40)).

After splitting the datasets to training and test sets, the Root Mean Squared Error (RMSE) values and scatter plots of actual versus predicted values and predicted values versus residuals (see Fig. S2) indicated that it performed consistently well: the RMSE was 5.03 and 9.69 for the pre-Omicron and the Omicron periods, respectively, and 9.87 for the overall COVID-19 data.

Removing the top 1% of exposure values in separate sensitivity analyses for the models Overall, Omicron period and pre-Omicron period did not substantially change the shape or strength of the exposure–response functions (see Figs. S3 and S4). This supports the robustness of the main findings to potential outliers or extreme exposure events.

## Discussion

Our analysis aimed to comprehensively investigate the associations between environmental stressors and COVID-19 over time, encompassing multiple stressors and demographic factors. The model incorporated temporal effects as independent variables. While non-pharmaceutical interventions (NPIs), immunization rates, population density, and circulating SARS-CoV-2 variants were not explicitly modeled, quarterly aggregation allows for partial adjustment for these changing factors. Postal code areas are included as MRF in the GAMs. The models included age group and sex assigned at birth as fixed effects to adjust for their potential confounding effect. Three models with the same structure but different time periods were fitted, the Overall period (2020–2022), pre-Omicron period (2020–2021) and Omicron period (2022). For the pre-Omicron period, the directions of association were broadly consistent with findings for influenza incidence before the COVID-19 pandemic^24^. In the Omicron period, these associations were entirely lost (Temp), or even reverted (PM_2.5_) (Table 1). It is an attractive hypothesis to ascribe the extensive loss of the association between environmental stressor and COVID-19 to the exaggerated virulence of the Omicron variant.

The Omicron variant is considered to be a particularly contagious and vaccine-resistant mutation^47–49^. During the period dominated by the Omicron variant, the main driver of the epidemic changed from contact rates to contagiousness^50^. Models suggest that the Omicron variant of SARS-CoV-2 may be up to 10 times more transmissible than the original strain and 2.8 times more transmissible than the Delta variant. Furthermore, it has been estimated that there is an 88% probability of Omicron evading the current vaccines^48^. On the other hand, the Omicron variant seems to result in less severe outcomes in terms of hospitalization, ventilation therapy, and death compared to previous variants^51–53^.

Most studies established a positive association between air pollutants, such as PM_2.5_, NO_2_, and O_3_, and respiratory diseases in general^54^, and COVID-19 in particular^2,3,7–9^. However, two review articles also indicated negative associations, as shown by Monoson^55^ and Carballo^3^.

A global meta-analysis of short-term exposure to air pollution discovered that COVID-19 incidence was positively associated with PM_2.5_^12^. A second meta-analysis found that 10 µg/m^3^ of PM_2·5_ increased odds of infection by 66%^56^. A review highlighted that most studies reviewed demonstrated a positive connection between COVID-19 infection and PM_2.5_ exposure to air pollution over both short and long term^9^. The positive association is also detected in the United Kingdom^57,58^ and Italy^13,59,60^. Additionally, a study in Germany revealed that each one-unit increase in PM_2·5_ resulted in almost 200 more cases of COVID-19 per 100,000 inhabitants by February 2021^22^. There is a biologically plausible mechanism that supports the high infection rate associated with PM_2.5_ exposure^61^. A positive correlation between NO_2_ and COVID-19 incidence was often found in the literature^4,9,12,59^, especially in Germany during the observation period of COVID-19 cases until September 2020^62^. On the other hand, two studies conducted in Italy indicated a negative correlation between NO_2_ levels and the incidence of COVID-19, at the beginning of the pandemic^20,63^. There is no clear association direction for O_3_ in the literature. Positive as well as negative associations between O_3_ exposure and COVID-19 incidence have been found^63–65^. A study across 409 cities in 26 countries also found little evidence of meteorological conditions like precipitation influencing COVID-19 transmission^66^. The literature has shown a link between higher temperatures and lower COVID-19 incidence rates, indicating a negative association^13–15^.

According to our COVID-19 models, the relative risk of contracting COVID-19 was lower for men than women, with a coefficient between 0.91 and 0.92 across all models. Northern Italy showed a similar RR value of 0.96 for men^60^. However, in general, men are thought to be more vulnerable to COVID-19 infections and more likely to experience severe symptoms of the disease^67,68^.

The above findings during the pre-Omicron period align with our model. COVID-19 incidence is positively associated with PM_2.5_ and negatively associated with Temp. Our model shows weak or no links with NO_2_, O_3_, and Prec, which are not clearly or distinctly described in the literature.

As far as we know, no study has been conducted on the relationship between the incidence of COVID-19 and environmental stressors covering the entire period from 2020 to 2022. Renard’s study examined the same time frame for Western Europe but with regard to COVID-19 mortality, reporting a positive correlation between short term exposure to PM_2.5_ and mortality^69^. However, our analysis focused on COVID-19 incidence. The overall model generally has similar trends to influenza incidence found from 2010 to 2018^24^. The literature classification is similar to the pre-Omicron period. The deviating positive association between incidence and O_3_ aligns with the literature mentioned.

To our knowledge, almost no studies have isolated the Omicron period from the preceding periods in terms of the relationship between COVID-19 incidence and environmental stressors. This is an area that requires further investigation in future studies. Only one study on the relationship between new infections and temperature in Verona up to and including July 2022 explicitly considered the Omicron period^70^. In this study, the previously observed negative correlation between temperature and the number of new infections is no longer observed due to the dominance of the Omicron variant. Instead, a positive correlation is observed. The paper discussed that the virus and its biological properties may have continuously changed over time. It was also suggested that the extremely high transmissibility of the virus could diminish the influence of environmental stressors.

Our study has high value due to its broad period, area-wide analysis, multiple stressors, demographic factors, high acquisition rate (notifiable disease in Germany)^71^, and differentiation between COVID-19 overall, Omicron, and pre-Omicron variants. To the best of our ability, our analysis incorporated Villeneuve’s commentary on methodological considerations for epidemiological studies of air pollution and COVID-19^72^. We introduced confounding variables such as age and sex and used high spatial resolution to ensure accuracy. Using an MRF smoother captures constant spatial structures, but not recorded time-varying, location-specific factors in postcode areas. As a result, potential spatial confounding factors may be unconsidered. While the GAM offers valuable insights, like any modeling approach, it approximates reality and cannot fully capture the complexity of real-world dynamics. A restriction of our analysis is the limited temporal resolution in quarters. This prevents us from examining potential short-term lag effects of environmental stressors on COVID-19 incidence; future studies with data of finer temporal resolution—when they become available—could explore this aspect in more detail. In addition, COVID-19 cases tend to be underestimated due to the dynamic situation regarding tests, doctor’s visits, and specific diagnoses. We excluded the NPI, the ratio of basic immunization, and SARS-CoV-2 variants due to their link to the quarter. Their exclusion avoided multicollinearities and improved model interpretation while indirectly retaining information via quarter. This study relies on observational data, which means that the identified associations should not be interpreted as causal relationships. Although we have adjusted for relevant covariates, factors that were not measured and potential reverse causality—such as pollution reductions resulting from mobility restrictions—could affect the observed associations. Our analysis was limited to COVID-19 incidence and cases. Only outdoor air pollution was considered. Future analysis may benefit from including the year 2023, which was also dominated by Omicron.

To study the impact of outdoor air pollution, we used the validated regional reanalysis from the Copernicus Atmosphere Monitoring Service (CAMS)^73^. Validation studies and continuous evaluation ensure its data quality. An evaluation study for Germany showed an underestimation of PM_2.5_ exposure compared to in-situ measurements^74^. Its spatial resolution represents background conditions but has proven reliable for air pollution and health risk assessment studies on small geographical areas^38^. At the time of our study, the CAMS regional reanalysis was found to be the most comprehensive data set available for capturing air pollution variability.

## Conclusion

The results obtained from our models highlight the complex association between environmental stressors and COVID-19 incidence. In the pre-Omicron period, the impact of these environmental stressors appears comparable to their known influence on influenza incidence. Higher levels of PM_2.5_ and O_3_ are linked to increased COVID-19 incidence, while temperature shows a negative association. In contrast, associations with Prec and NO_2_ are weak. During the Omicron period however, these associations change, particularly for Temp, PM_2.5_ and O_3_. All stressors, with the exception of O_3_, show weak or no clear cut-off association with COVID-19 incidence. Notably, the effects of O_3_ reverses to a negative association. This change may reflect Omicron’s increased infectiousness and transmissibility, potentially reducing the response to environmental stressors. These findings nourish the hypothesis that Omicron is sufficiently contagious that environmental factors no longer have a measurable impact on COVID-19 incidence. In contrast, during the pre-Omicron period, reductions in air pollution might have contributed to lowering COVID-19 transmission, as has been observed for other respiratory infections.

## Supplementary Information

Supplementary Information.

## Data Availability

The data that support the findings of this study are available from the corresponding author upon reasonable request.

## Abbreviations

AOK: Allgemeine Ortskrankenkasse
BW: Baden-Württemberg
C3S: Copernicus climate change service
CAMS: Copernicus atmosphere monitoring service
CI: Confidence interval
COVID-19: Coronavirus disease of 2019
DLR: German aerospace center
ECMWF: European centre of medium-range weather forecasts
ESA: European space agency
GAM: Generalized additive model
ICD-10: International classification of diseases 10th revision
IMBIE: Institute of medical biometry, informatics and epidemiology
NO_2_: Nitrogene dioxide
NPI: Non-pharmaceutical intervention
O_3_: Ozone
PM_2.5_: Fine particulate matter with a diameter of 2.5 µm or smaller
Prec: Precipitation
RKI: Robert Koch Institute
RR: Relative risk
SARS-CoV-2: Severe acute respiratory syndrome coronavirus 2
Temp: Temperature
