# Supplementary material for: Modulation of COVID-19 incidence by environmental stressors is variant between pre-Omicron and Omicron periods
Source: Sci Rep. 2025 Jul 29;15:27636. doi: 10.1038/s41598-025-13521-2 (PMC12307877; doi:10.1038/s41598-025-13521-2)
Supplement: Supplementary file 1 — Supplementary Information. [file 41598_2025_13521_MOESM1_ESM.docx]

# Supplemented Material

# Modulation of COVID-19 incidence by environmental stressors is variant between pre-Omicron and Omicron periods

Leona Hoffmann^1*^, Lorenza Gilardi^2^, Tobias Antoni^3^, Maxana Baltruweit^3^, Michael Bittner^2^, Susanne Breitner^4,5^, Simon Dally^3^, Thilo Erbertseder^2^, Sabine Hawighorst-Knapstein^3^, Marie-Therese Schmitz^6^, Rochelle Schneider^7^, Sabine Wüst^2^, Jörn Rittweger^1,8^

^1^Institute of Aerospace Medicine, German Aerospace Center (DLR), Cologne, Germany

^2^German Remote Sensing Data Center, German Aerospace Center (DLR), Weßling, Germany

^3^Allgemeine Ortskrankenkasse Baden-Württemberg (AOK-BW), Stuttgart, Germany

^4^ Institute of Epidemiology, Helmholtz Zentrum München - German Research Centre for Environmental Health, Neuherberg, Germany

^5^ IBE-Chair of Epidemiology, LMU Munich, Munich, Germany.

^6^Institute of Medical Biometry, Informatics and Epidemiology (IMBIE), University Hospital Bonn, Bonn, Germany

^7^Φ-lab, European Space Agency (ESA), Frascati, Italy

^8^Department of Pediatrics and Adolescent Medicine, University Hospital Cologne, Cologne, Germany

^*^Corresponding author. Email: leona.hoffmann@dlr.de

|  | **Statistical Parameter** | **2020** | **2021** | **2022** |
| --- | --- | --- | --- | --- |
| **PM_2_**_·_**_5_** | Mean (SD)  Median [Min, Max] | 8.43 (1.07)  8.39 [5.25, 12.1] | 8.89 (1.34)  8.49 [6.21, 12.0] | 8.51 (2.08)  7.84 [5.30, 14.3] |
| **NO_2_** | Mean (SD)  Median [Min, Max] | 11.1 (4.48)  10.6 [2.59, 23.7] | 10.5 (4.53)  9.73 [2.77, 22.9] | 9.34 (3.97)  8.75 [2.78, 20.5] |
| **O_3_** | Mean (SD)  Median [Min, Max] | 52.8 (16.3)  58.3 [20.3, 81.2] | 52.6 (13.5)  53.1 [24.9, 78.6] | 58.1 (16.5)  65.4 [23.6, 87.1] |
| **Temp** | Mean (SD)  Median [Min, Max] | 10.6 (5.89)  9.74 [2.09, 21.1] | 9.02 (5.61)  8.10 [0.36, 18.5] | 10.9 (5.91)  10.7 [1.56, 20.9] |
| **Prec** | Mean (SD)  Median [Min, Max] | 3.24 (0.76)  3.17 [1.45, 6.16] | 3.85 (0.84)  3.63 [2.36, 6.98] | 2.98 (0.66)  2.74 [1.68, 5.65] |

**Table S1:** **Overview of environmental stressors.** Overview of meteorological data and air pollutants, including statistical parameters mean, standard deviation (SD), median, minimum (min), and maximum (max) values. The data cover all postal code areas in BW and are based on quarterly measurements taken from 2020 to 2022.

**
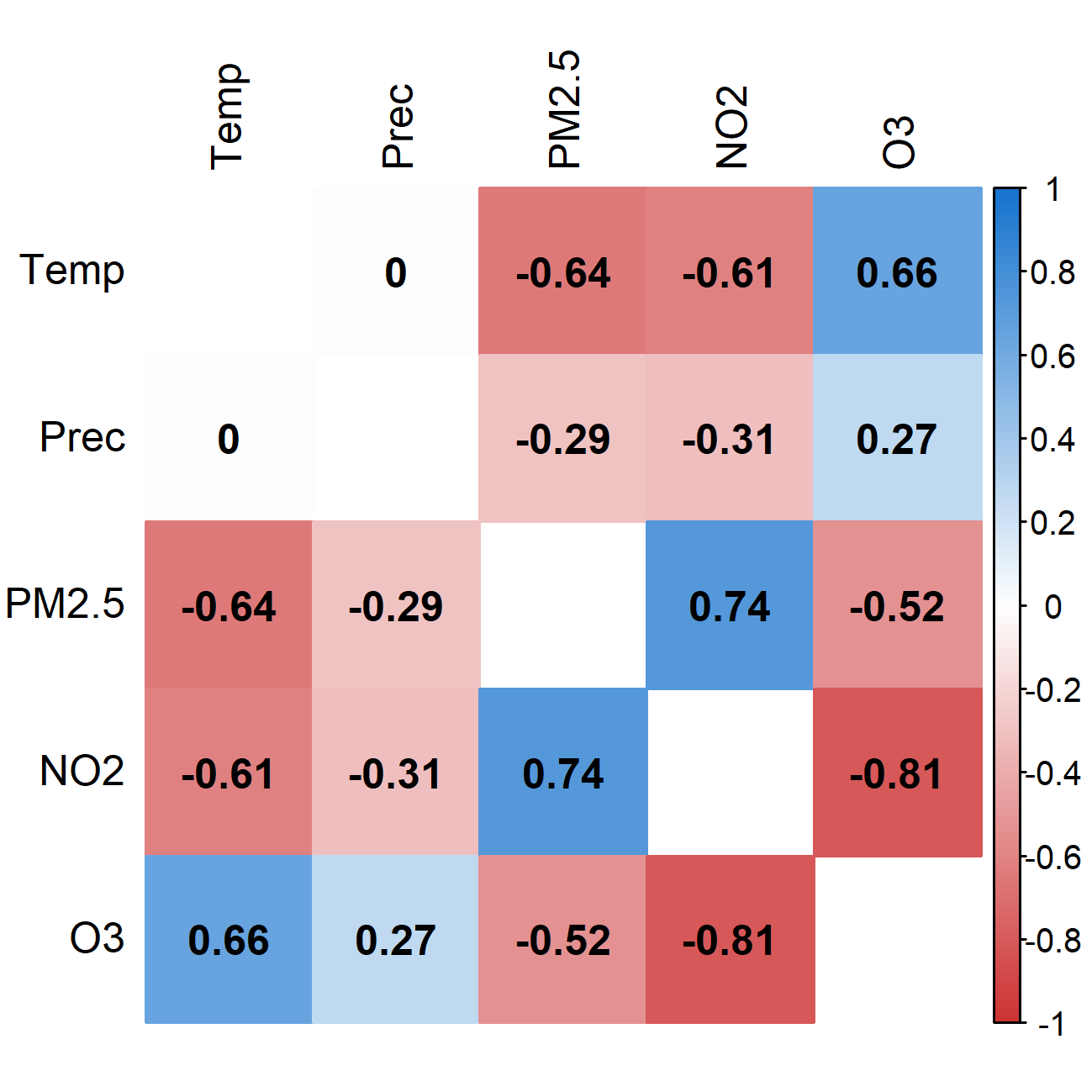
Figure S1:** **Pearson correlation matrix based on quarterly measurements from 2020 to 2022 across the German federal state Baden-Württemberg (BW).** The intensity of the color indicates the strength of the correlation between the two variables, with blue indicating positive correlations and red indicating negative correlations.


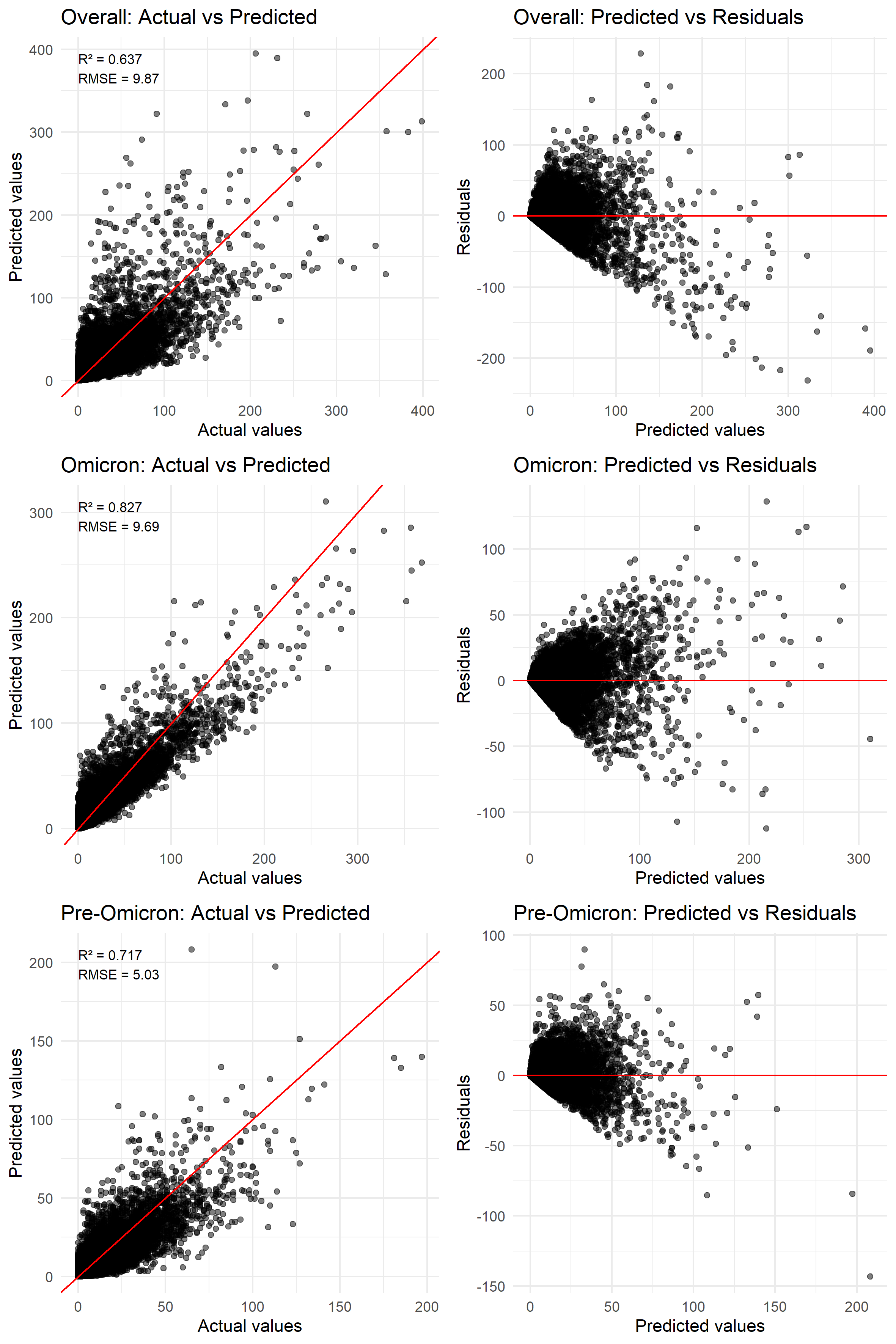


**Figure S2: Scatter plots of actual versus predicted values and predicted values versus residuals for all three models: Overall, Omicron and pre-Omicron period.** Additional, R² and RMSE are provided.


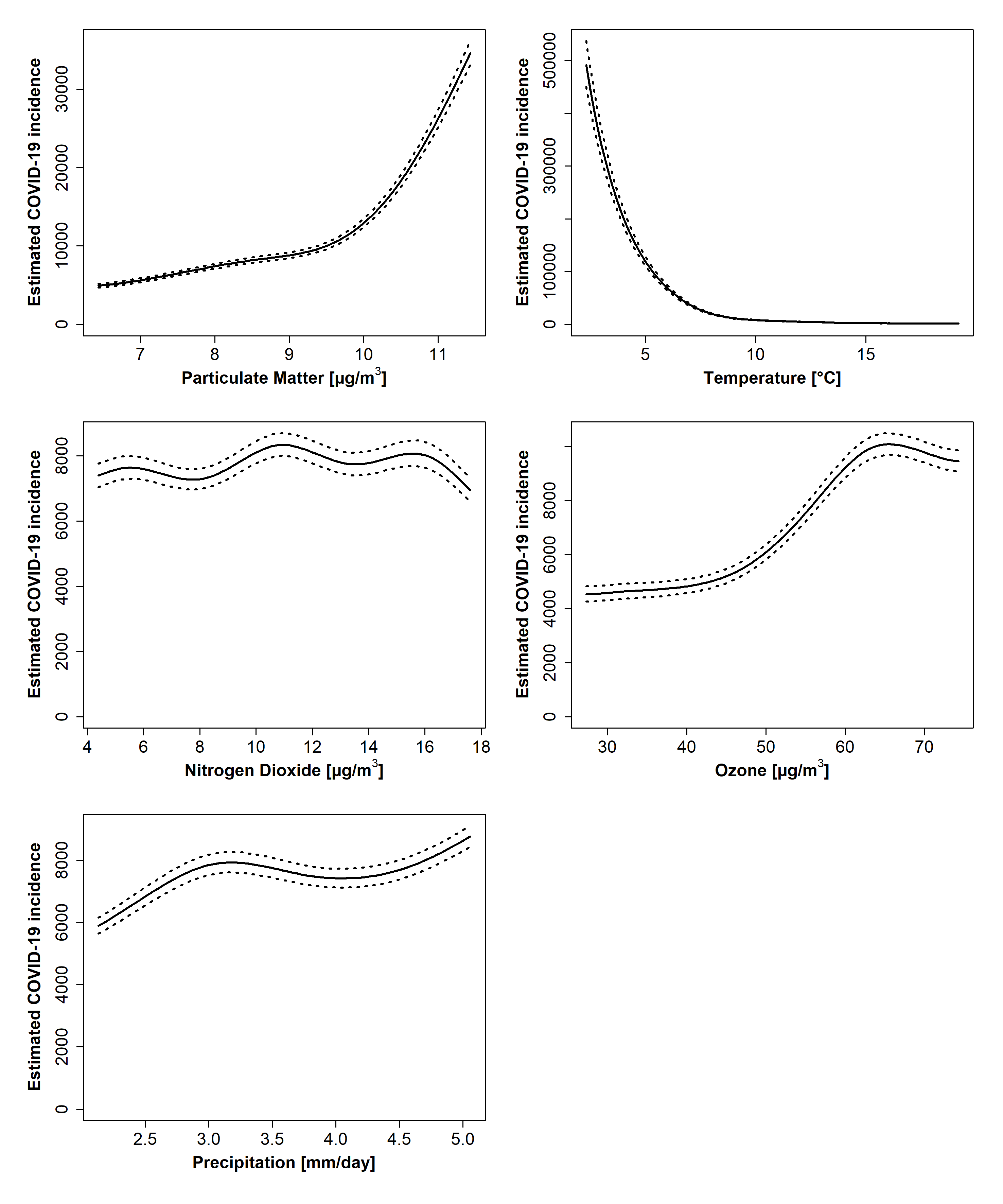


**Figure S3:** **Sensitivity analyses excluding extreme pollution values (top 1%): Estimated COVID-19 incidence per 100,000 persons per year in response to each environmental stressor.** All other parameters were kept constant, and were set to the following values: Age group: 50-60 years, sex assigned at birth: female, postal code: 70376, quarter: Q2, year: 2021 and environmental stressors: median. Estimates were obtained using the R function predict for Temp, Prec, NO_2_, O_3_, and PM_2·5_, including their 5th and 95th percentiles.


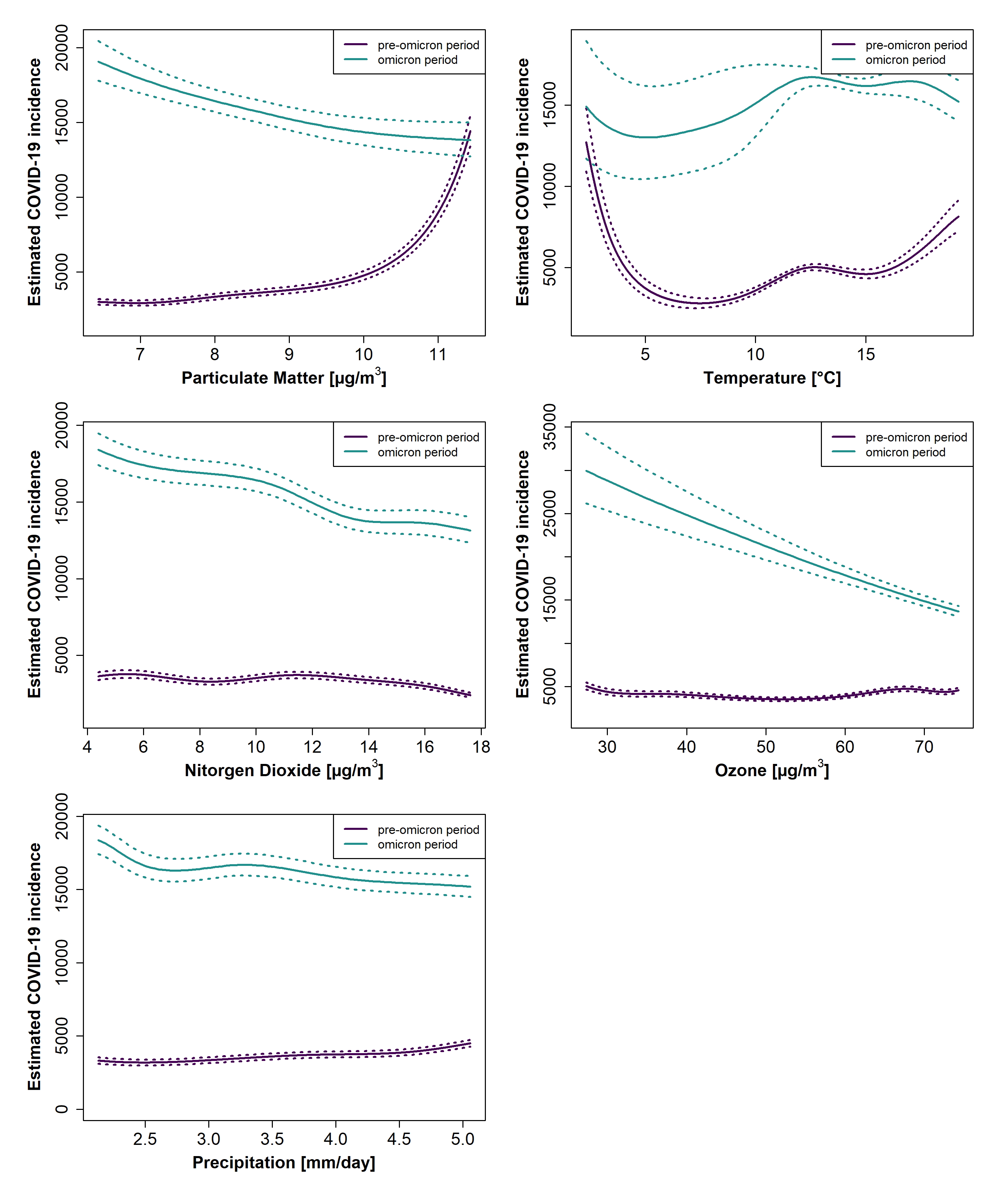


**Figure S4:** **Sensitivity analyses excluding extreme pollution values (top 1%): Estimated COVID-19 incidence per 100,000 persons per year in response to each environmental stressor based on pre-Omicron (2020, 2021, purple) and Omicron (2022, teal) model.** All other parameters were kept constant and were set to the following values: quarter: Q2, year (optional): 2021, postal code: 70376, sex assigned at birth: female, age group: 50 to 60 years, and environmental stressor: median. Estimates were obtained using the R function predict for Temp, Prec, NO_2_, O_3_, and PM_2·5_, including their 5th and 95th percentiles.
